# Identification and characterization of miRNAome and target genes in *Pseudostellaria heterophylla*

**DOI:** 10.1371/journal.pone.0275566

**Published:** 2022-10-05

**Authors:** Jun Li, Chongmin Wang, Tao Zhou, Haijun Jin, Xiaoqing Liu

**Affiliations:** Guizhou University of Traditional Chinese Medicine, Guiyang, China; Birla Institute of Technology and Science, INDIA

## Abstract

miRNAs play a crucial role in the development and growth of plants by inhibiting the function of targeted genes at the post-transcription level. However, no miRNAs in *Pseudostellaria heterophylla* have been reported and their function in the morphogenesis of organs is still unclear. In this study, a total of 159 conserved miRNAs (belonging to 64 families) and 303 level miRNAs were identified from *P*. *heterophylla*. Some of them showed specifically up or down-regulated expression in different tissues and numbers of unigenes involved in Plant-pathogen interaction and MAPK signaling pathway-plant were targeted. The significant negative correlation of expression profiles between 30 miRNAs and their target genes (37 unigenes) was observed, respectively. Further, a large number of genes involved with signal transduction of auxin, zeatin, abscisic acid and, jasmonic acid were targeted. Predicated targets of two miRNAs were validated by 5′RLM-RACE, respectively. A large number of mRNAs from four pathogens were targeted by miRNAs from *P*. *heterophylla* and some of them were targeted by miR414. In summary, we reported a population of miRNAs from four different vegetative tissues of *P*. *heterophylla* by high throughput sequencing, which was analyzed by combining with the constructed transcriptome. These results may help to explain the function of miRNAs in the morphogenesis of organs and defense of pathogens, and may provide theoretical basis for breeding and genetic improvement of *P*. *heterophylla*.

## Introduction

MicroRNA (miRNA) is a kind of endogenous single-stranded non-coding small RNA [1], which inhibits the function of targeted genes at the post-transcription level by degrading or inhibiting the translation [2]. Since the first miRNA (lin 4) in *C*.*elegans* was identified [3], a large number of miRNAs have been discovered from various species. Recent evidence has indicated that miRNA can not only impact the growth and development of plants, and the differentiation and morphogenesis of organs by directly regulating the target genes [4], but also participate in the signal transduction of plants [5]. miRNAs can also improve the tolerance to environmental stress by regulating the transcription factor in plants [6], such as drought, low temperature, high salt, and low nitrogen. Furthermore, miRNAs may improve the resistance to biological stress of plants by inhibiting the gene expression of pathogenic microorganisms [7, 8].

During the synthesis of miRNAs, primary miRNAs (pre-miRNA) are firstly transcribed by RNA polymerase II [9]. The pre-miRNAs are processed into ∼70-nt premiRNAs by Dicer-like protein in association with other protein factors (HYL1and others) and subsequently released as miRNA: miRNA* duplexes by different DCL protrein [10]. The 3′ terminal of these duplexes is methylated by HEN1, which can prevent miRNA degradation and help the miRNA be incorporated into the silencing complex or effector complex (RISC-RNA Induced Silencing Complex) containing argonaute proteins [11–13]. Methylated miRNA: miRNA* duplexes are subsequently transported to the cytoplasm with the help of the HASTY protein [11, 14], while the other strand (miRNA* or passenger strand) gets degraded. The RISC-RNA-induced silencing complex reduces the expression of target mRNA by guiding the cleavage of complementary target mRNAs and inhibiting its translation [13, 15]. Under the new naming rules, new miRNA is no longer represented by miRNA and miRNA*, but named miRNA -3p and miRNA -5p based on the position of the mature miRNA in the precursor [16]. miRNA -3p is close to the 3′end of the precursor and miRNA -5p is close to the 5′end of the precursor [17].

A large number of miRNAs are evolutionary conserved among diverse species in the plant kingdom with conserved regulation mechanisms, such as the expression of a class III homeodomain-leucine zipper (HD-Zip III) protein and miR166, APETALA2, and miR172 [18]. Previous studies have shown that several miRNAs had species-specificity in each plant and may participate in organ morphogenesis and defense response. However, most of the species-specific miRNAs remained unidentified in many plants because of their low expression [18, 19]. In recent decades, species-specific miRNAs have been identified in diverse plant species with the advent of high-throughput sequencing technology [20–23]. Furthermore, it is possible to identify new miRNAs from different tissues at different developmental stages [24].

*Pseudostellaria heterophylla* is a kind of Chinese medicinal material widely distributed in China. Tuberous root of *P*. *heterophylla* was called *Pseudostellariae* Radix, which contains polysaccharides, saponins, phospholipids, cyclopeptides, fatty acids, and volatile components [25]. *Pseudostellariae* Radix was commonly used for the protection of myocardial, enhancement of immunity, anti-oxidation, hypoglycemia, and anti-fatigue [26]. Although there were a lot of studies on the chemical composition and pharmacological action of *P*. *heterophylla* in recent years [27, 28], the exploration of the regulation mechanism of plant growth and continuous cropping obstacles is still needed. In previous studies, a transcriptome database of *P*. *heterophylla* has been constructed from different tissues [29]. In this study, we report a population of *P*. *heterophylla* miRNAs from vegetative tissues (leaf, stem, xylem and bark of tuberous root). The species-specific miRNAs were both identified by searching the newly constructed transcriptome of *P*. *heterophylla*. The expression correlation between miRNAs and their targets was analyzed by combined with the transcriptome. Gene Ontology (GO) and the Kyoto Encyclopedia of Genes and Genomes (KEGG) were used for exploring the functions of the predicted targets. The target genes of two miRNAs were validated through 5′RLM-RACE. Reported mRNA sequences of 4 pathogens were downloaded from GenBank and used for searching the target genes of miRNAs from *P*. *heterophylla*. These results may help to explain the function of miRNAs in morphogenesis of organs and defense of pathogens in *P*. *heterophylla*, and may also provide theoretical basis for breeding and genetic improvement of *P*. *heterophylla*.

## Results

### An overview of high-throughput sequencing data sets

To identify the miRNAs in *P*. *heterophylla*, cDNA library and small RNA library from four tissues (leaves, stem, cortex, and xylem of the tuberous root) were constructed, respectively. In the cDNA library, we obtained 603,398,664 raw reads and 598,156,596 clean reads from four tissues with the base average error rate below 0.02%. Trinity program was used for *de novo* assembling clean data. 138,888 unigenes and 207,390 transcripts were obtained. The length of unigenes ranged from 201 to 14,793 bp and the N50 length was 1,862. 71,215 unigenes (51.28%) and 121,508 transcripts (58.59%) were annotated based on the public databases.

**Fig 1:**
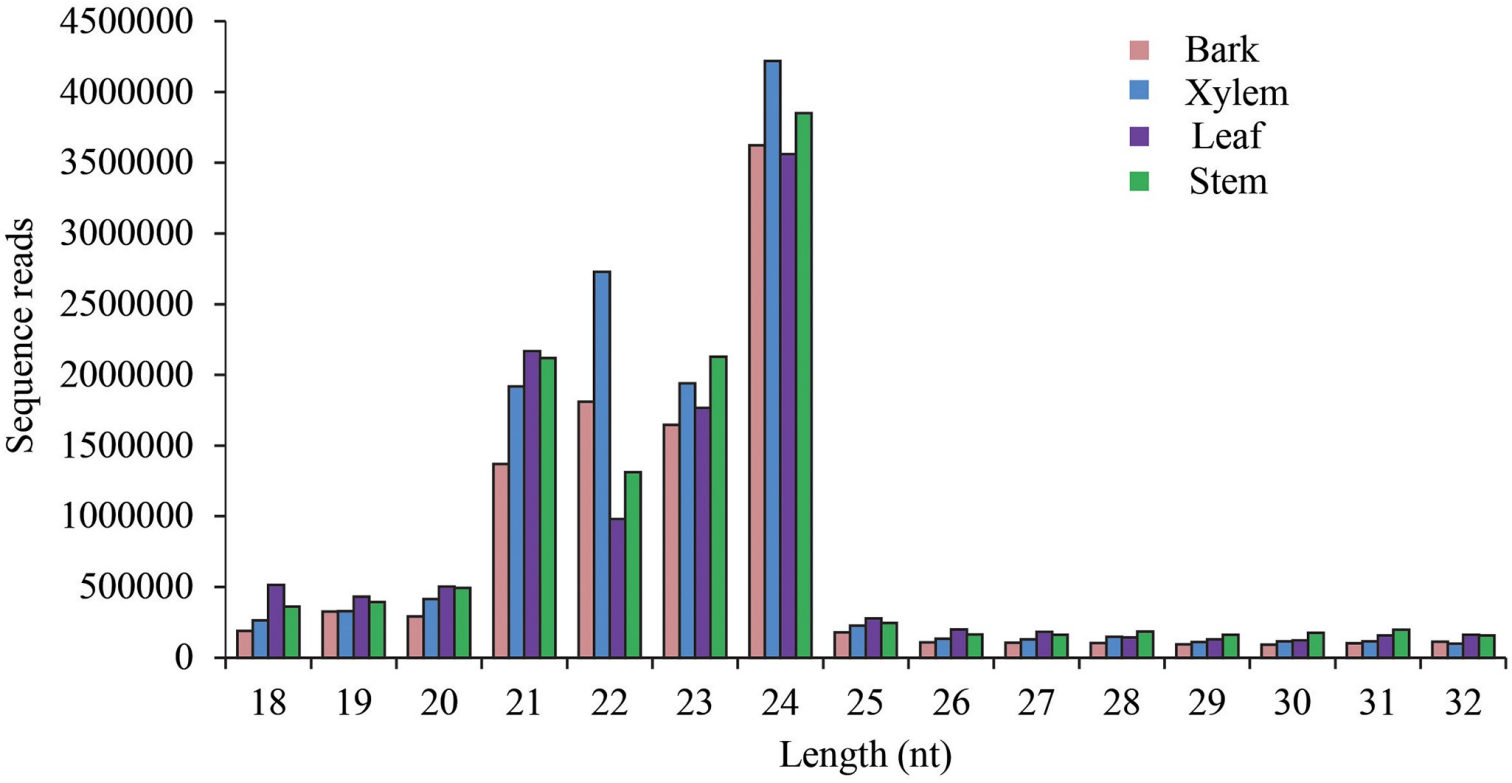
Size distribution of sRNAs from the leaves, stem, bark, and xylem of tuberous root in *P*. *heterophylla*.

In the small RNA library, a total of 180 million raw reads and 139 million clean reads were yielded from these 12 libraries (Table 1). These raw data were filtered through several criteria to identify conserved and specific miRNAs. The length distribution of the small RNAs was analyzed and shown in Fig 1. The majority of reads were 21 to 24 nt in length. The 24-nt RNA is the most abundant class in the sRNA library and comprised 35.7% of root bark, 32.7% of root xylem, 31.5% of leaf and 31.8% of stem, respectively. Similar results were reported in potato [24] and cucumber [30], but in contrast to wheat [31].

**Table 1:** Summary of miRNAs from *P*. *heterophylla*.

Tissues	Samples	Total_Reads	Total_Bases	Clean_reads	<18nt	>32nt	Error%	Q20%	Q30%	GC%
**Leaf**	L_1	19997062	1019850162	14687858	4489811	641717	0.0096	99.53	98.32	49.86
	L_2	15723616	801904416	11662446	3205875	766081	0.0096	99.51	98.26	49.45
	L_3	11719745	597706995	7532181	3454592	666546	0.0094	99.56	98.43	50.3
**Stem**	S_1	17746249	905058699	13497506	3639872	510474	0.0094	99.58	98.49	49.96
	S_2	14178512	723104112	10361306	3127814	632337	0.0094	99.6	98.53	51.17
	S_3	15462167	788570517	12465662	2123694	816294	0.0094	99.59	98.53	49.98
**Xylem of tuberous root**	X_1	14109579	719588529	11751750	1497786	805939	0.0098	99.42	97.88	48.83
	X_2	15962140	814069140	13530278	1872942	464097	0.0096	99.51	98.24	48.81
	X_3	16907101	862262151	13375075	2649887	762616	0.0095	99.56	98.37	49.39
**Bark of tuberous root**	B_1	13331179	679890129	10538460	1754871	986394	0.0098	99.44	97.93	48.79
	B_2	12164286	620378586	9618731	1674710	815536	0.0097	99.49	98.08	49.12
	B_3	13025482	664299582	10284639	1416905	1272697	0.0098	99.42	97.88	48.54

### Identification of known miRNAs in *P*. *heterophylla*

**Fig 2:**
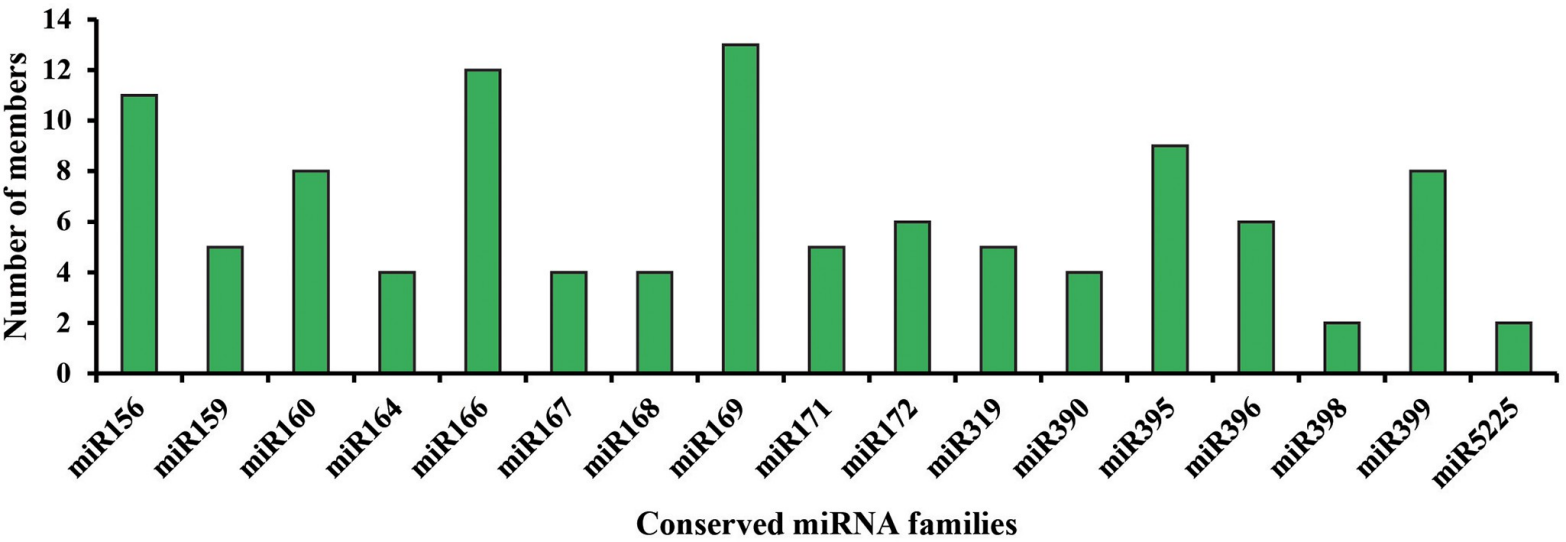
Conserved miRNA families and their members identified in *P*. *heterophylla*. Graphical representation of the different members of conserved miRNA families identified from leaves, stem, bark, and xylem of tuberous root in *P*. *heterophylla*.

In order to identify the conserved miRNAs in *P*. *heterophylla*, the small RNA was submitted to the miRBase 22.1 (http://www.mirbase.org/). Sequences with high identifies to other species were considered as known miRNAs. A total of 159 miRNAs belonging to 64 families were identified (S1 Table). Among these families, miRNA169 comprised 13 members defined by distinguished based on nucleotide differences and followed by miRNA166 (12 members), miRNA156 (11 members), miRNA395 (9 members), miRNA160 (8 members), miRNA399 (8 members) (Fig 2). miRNA396, miRNA159, miRNA319, and miRNA166 showed high abundance with total TPM >100,000. The highest read abundance (7,202,956 normalized reads) was detected for miR396. However, 37 miRNAs with total TPM of less than 10 were found, such as miR1871, miR829, miR828, and miR2873.

### Identification of novel candidate miRNAs in *P*. *heterophylla*

**Fig 3:**
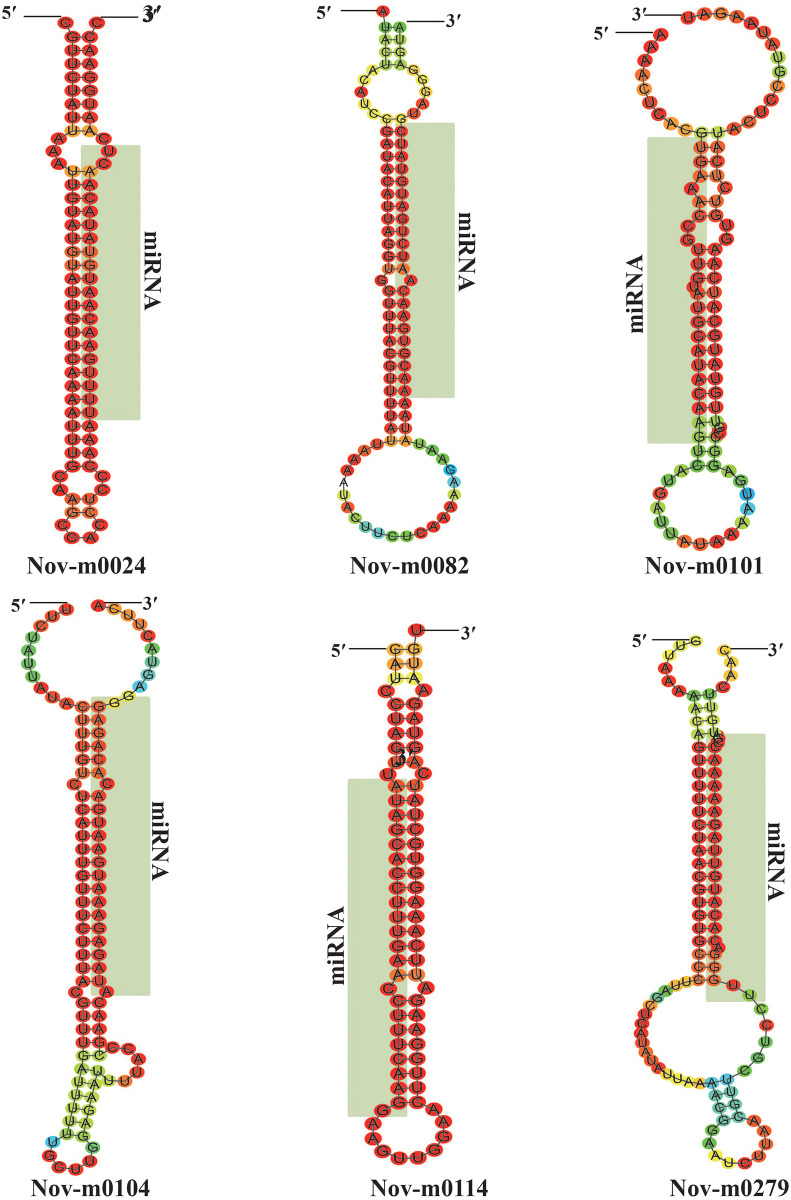
Predicted secondary structure of precursors (Mfold) of a few specific miRNAs of *P*. *heterophylla*. The mature miRNA sequences are highlighted with light green color.

The non-conserved small RNA reads were mapped to unigenes in the transcriptome of *P*. *heterophylla* and a total of 303 novel miRNA candidates were identified (S2 Table). A standard stem-loop hairpin secondary structure (SS) was found in all miRNA precursors (not shown). The secondary structure of precursors of novel miRNAs was predicted by using the Mfold web server [32] and a few were shown in Fig 3. These miRNA precursors had folding free energies ranging from -48 to -18 kcal/mol (average, -27.28 kcal/mol). The length of the predicted precursor of these novel miRNAs was 58–101 nt. The MFEI values of all miRNAs were above 0.85 and these sequences were considered as miRNAs [33]. 4 novel miRNAs had high expression level with TPM >1 000 and the highest read abundance (2 662 normalized reads) was detected for Phn-m0190. 73 novel miRNAs with total TPM of less than 10 were found, such as Phn-m0032, Phn-m0045, Phn-m0050, Phn-m0058, and Phn-m0076.

### Prediction of miRNA target genes

To explore the biological functions of identified miRNAs, the Target Finder program was used for searching the complimentary mRNA sequences from the transcriptome of *P*. *heterophylla* above. Among the conserved targets, numbers of unigenes coding transcription factors were identified (S3 Table), such as GAMYB-like (targeted by miR159), APETALA2 (targeted by miR172), PHAVOLUTA-like HD-ZIPIII protein (targeted by miR166), nuclear transcription factors YA3 and YA5, Zinc finger CCCH domain-containing protein and WRKY (targeted by miR169), NAC domain-containing protein (targeted by miR164), and squamosa promoter-binding protein (targeted by miR156). For novel miRNAs, 305 potential target genes involving protein kinase were identified (S4 Table). 162 potential target unigenes coding transcription factors were also observed, including MYB (44 unigenes), bHLH (34 unigenes), WRKY (15 unigenes), and MADS (12 unigenes). In addition, 34 potential target genes were founded to be involved in the synthesis and biotransformation of NADPH.

Multiple potential regulation patterns were discovered in several unigenes (S5 Table). 8 binding sites of Phn-m051-5p and Phn-m052-5p were identified in the mRNA sequence of ubiquitin-conjugating enzyme E2 24. Transmembrane protein 97 has 11 potential regulation sites targeted by miRNA399 families. Three HD-ZIPIII genes (ATHB8, ATHB15 and REVOLUTA) also have 9 potential regulation sites targeted by miRNA166 families. Furthermore, several unigenes were only targeted by novel miRNAs, such as multicopper oxidase LPR2, L-ascorbate oxidase homolog, ribosomal RNA-processing protein 7 homolog A and pyrrolidone-carboxylate peptidase. In addition, plenty of non-coding unigenes were targeted (S3 and S4 Tables), including 609 non-coding unigenes targeted by 89 conserved miRNAs and 3673 non-coding unigenes targeted by 274 novel miRNAs. These results indicated that miRNAs may also participate in the regulation of non-coding unigenes.

### GO and KEGG analysis

**Fig 4:**
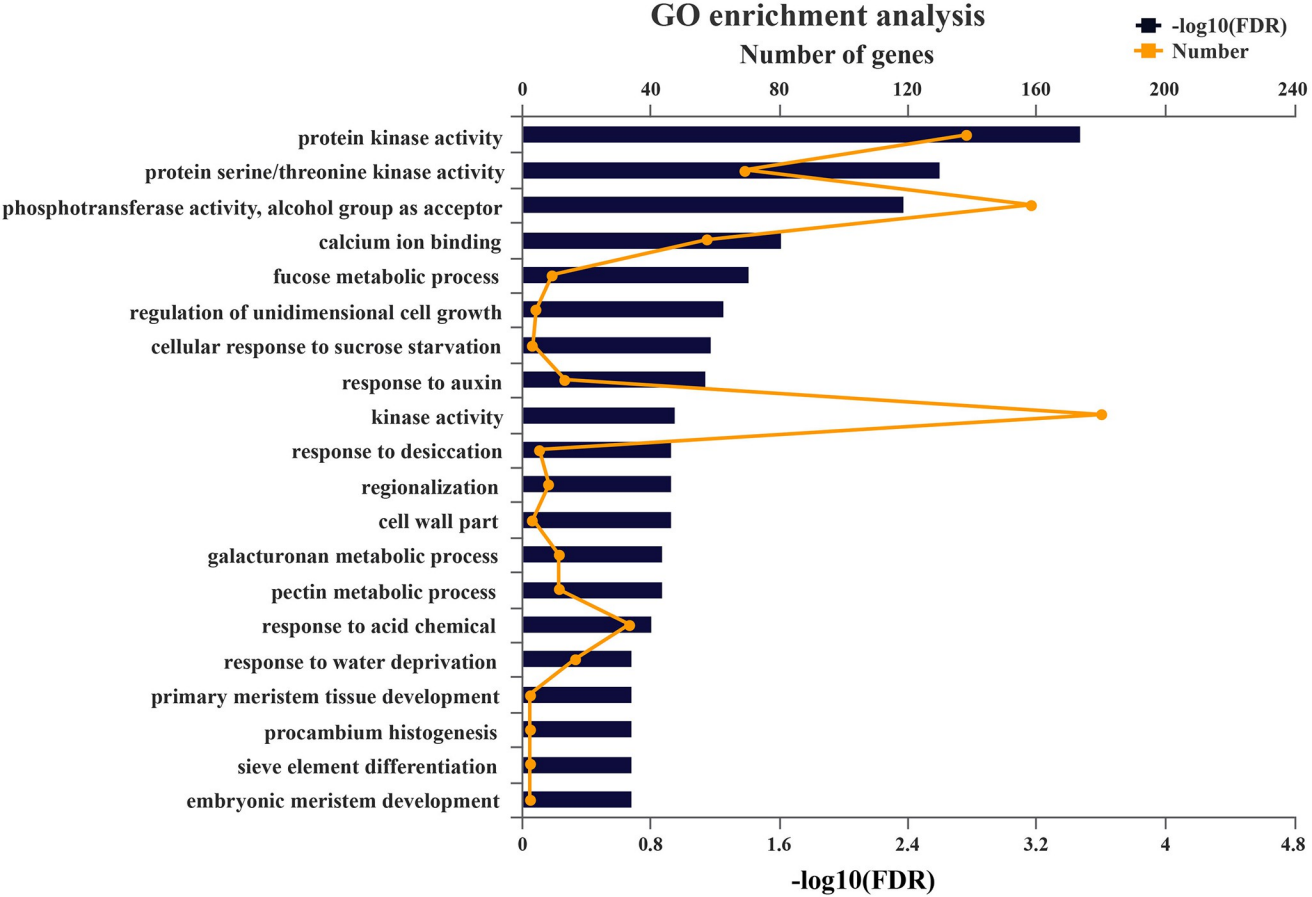
GO analysis of predicted putative target unigenes of miRNAs. The number of unigenes matched by the broken line. The significance of the matched gene ratio was shown by the column length of FDR.

**Fig 5:**
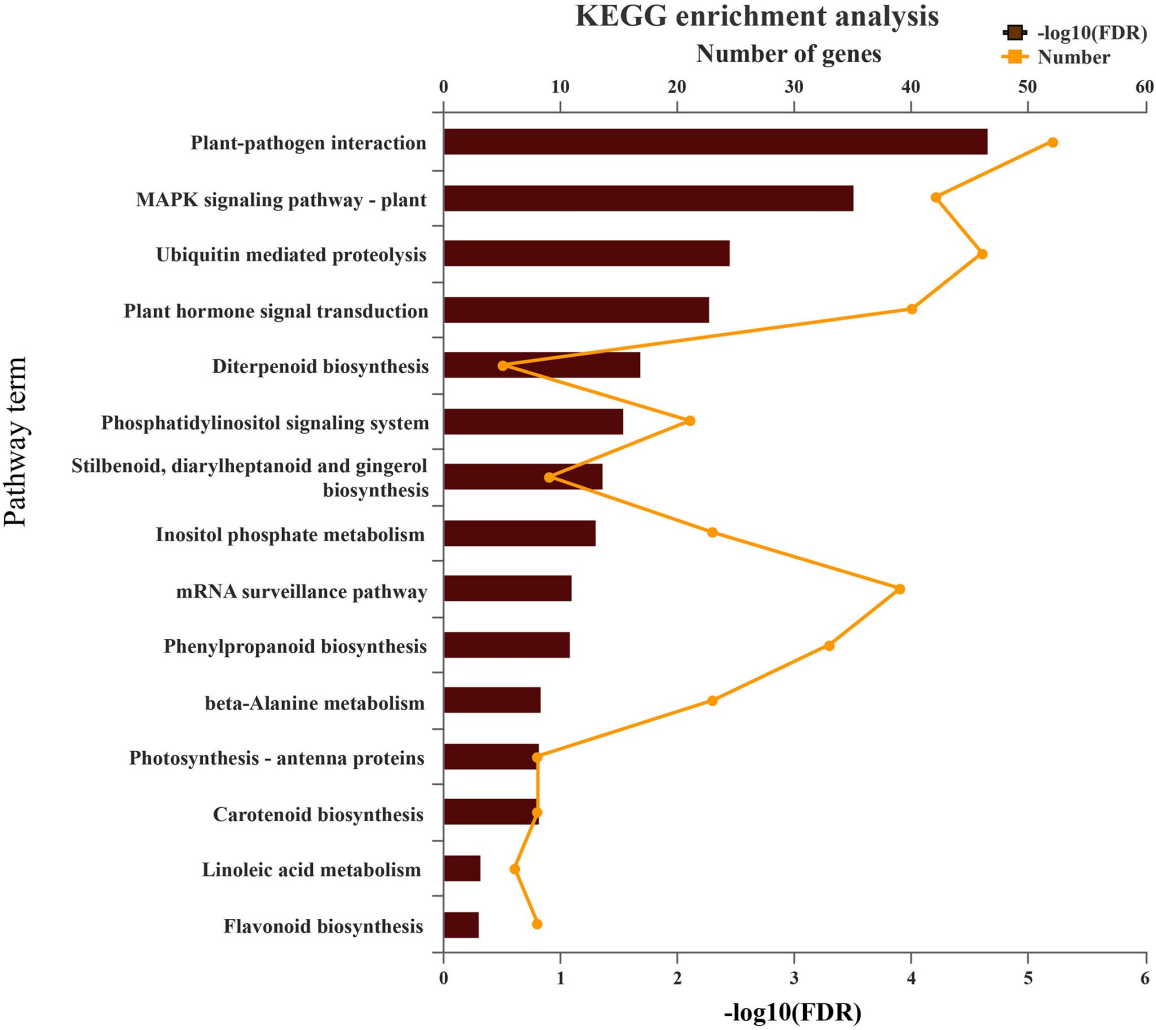
KEGG analysis of pathway enrichment for predicted putative target unigenes of miRNAs in the *P*. *heterophylla*. The number of unigenes matched by the broken line. The significance of the matched gene ratio was shown by the column length of FDR.

GO analysis was performed to explore the potential regulation of predicted targeted unigenes of miRNAs in *P*. *heterophylla*. Based on their functional annotations, the targeted genes were divided into 122 categories (S6 Table). In particular, 6 categories have higher enrichment, such as protein kinase activity, protein serine/threonine kinase activity, phosphotransferase activity, alcohol group as acceptor, calcium ion binding, kinase activity, and response to acid chemical (Fig 4). KEGG pathway enrichment analysis showed that the targeted genes of the miRNAs were mainly involved in 8 pathways, including Plant-pathogen interaction, MAPK signaling pathway-plant, plant hormone signal transduction, ubiquitin-mediated proteolysis, phosphatidylinositol signaling system, phenylpropanoid biosynthesis, inositol phosphate metabolism and diterpenoid biosynthesis (S7 Table and Fig 5). Numbers of targeted genes only involving in Plant-pathogen interaction were targeted, including 9 calcium-dependent protein kinases, 4 pto-interacting protein, 3 disease resistance proteins and 2 calcium-binding proteins, 2 cyclic nucleotide-gated ion channels. Several targeted genes were annotated only in MAPK signaling pathway-plant pathways, such as mitogen-activated protein kinase (MPK1, 3, 4, 8) and reversion-to-ethylene sensitivity (TMEM222). However, partial targeted genes participated in both Plant-pathogen interaction and MAPK signaling pathway-plant pathway, including 6 calmodulins, 6 respiratory burst oxidase homolog proteins, and 3 WRKY transcription factor. These results suggested that miRNAs in *P*. *heterophylla* may play an important role in the regulation of cell signal transduction.

### Role of miRNAs in terrestrial and underground tissues

**Fig 6:**
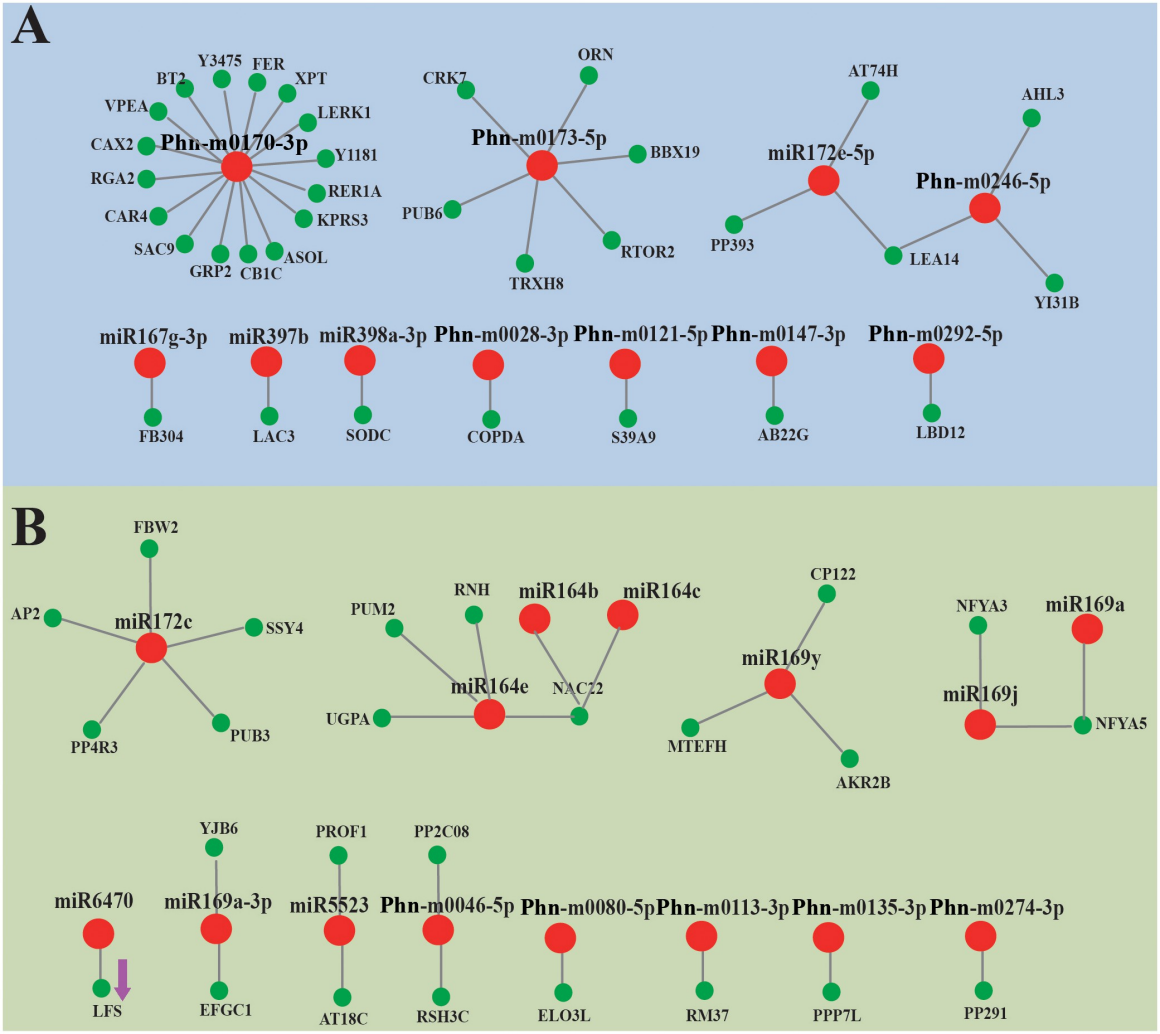
Tissue-special miRNAs in terrestrial tissues and underground tissues and their target genes. (A) A combined view of miRNAs and their targets in terrestrial tissues; (B) A combined view of miRNAs and their targets in underground tissues.

For a better understanding of the role of miRNAs in the morphogenesis of organs, tissue-special miRNAs were identified according to their expression profiles (S1 and S2 Tables), and correlation analyses were performed by combining with the transcriptome (S8 Table). 6 conserved miRNAs and 9 novel miRNAs was found to be up-regulated in underground tissues (root bark and xylem) and down-regulated in terrestrial tissues (stem and leaf). 115 potential target genes were identified (S9 Table), including 33 coding unigenes and 82 non-coding unigenes. 16 unigenes targeted by Phn-m0170-3p and 6 genes targeted by Phn-m0173-5p were identified. CRK7, PUB6, and BBX19 showed significantly negative correlation with the expression of Phn-m0173-5p. SODC may be negatively regulated by miR398a-3p. 12 conserved miRNAs and 13 novel miRNAs showed abundant expression in underground tissues while being restricted to terrestrial tissues. 107 potential target genes were identified, including 37 coding mRNA and 70 non-coding mRNA. Two conserved miRNAs (miRNA169a and miRNA169a) and a conserved miRNA (Phn-m0046-5p) were negatively correlated with NF-YA5 and PP2C08 respectively (Fig 6), which suggested their gene-silencing function.

### Role of miRNAs in the development of leaf

**Fig 7:**
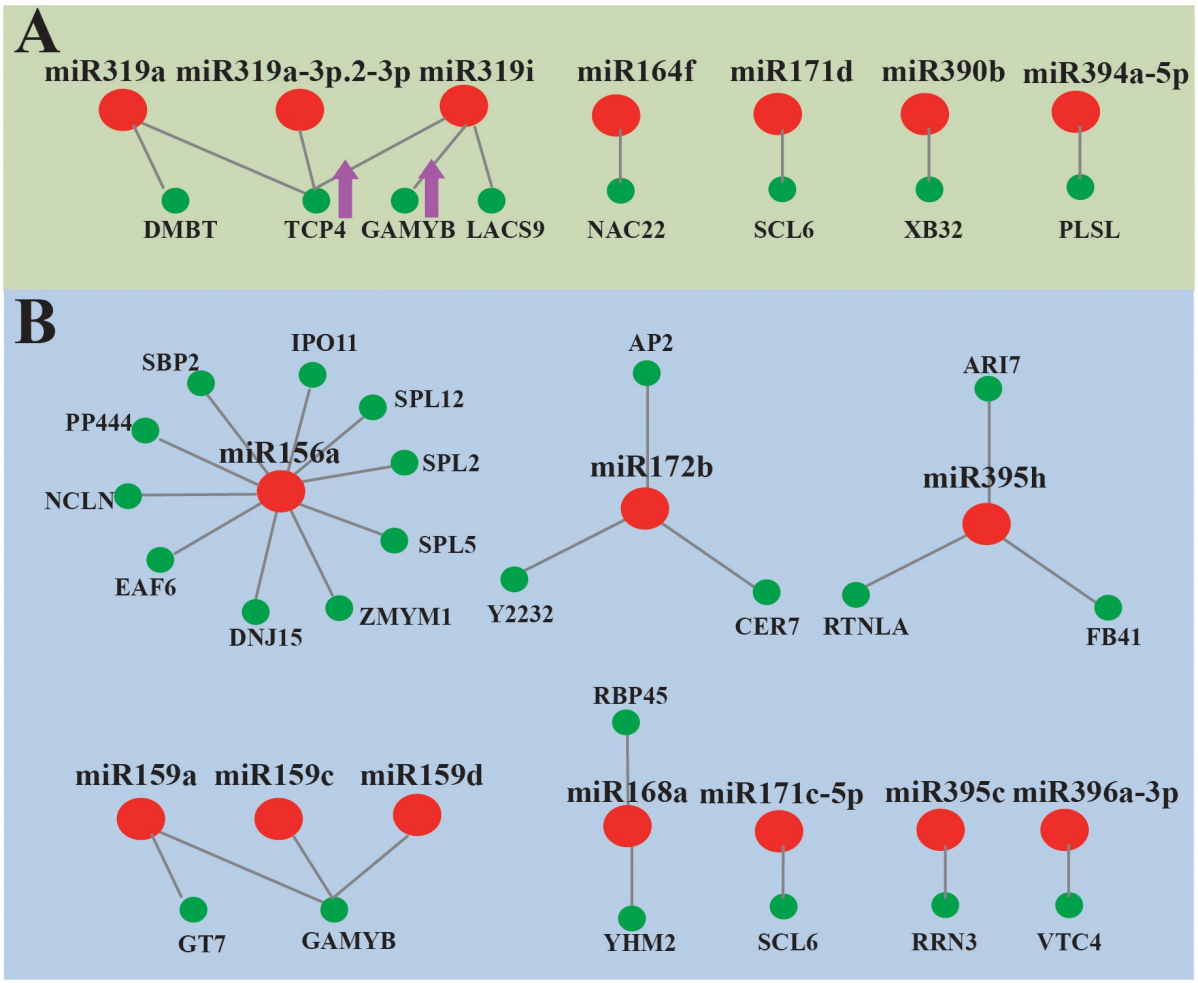
Leaf-special miRNAs and their target genes. (A) A combined view of down-regulated miRNAs and their targets; (B) A combined view of up-regulated miRNAs and their targets. Pink tip represents significant negative correlation.

9 conserved miRNAs and 2 novel miRNAs were significantly down-regulated in leaf (S10 Table). 36 potential target genes were identified, including 13 coding mRNA and 23 non-coding mRNA. TCP showed significantly negative correlation with 3 miRNA319 members, miR319a, miR319a-3p.2-3p, and miR319i (Fig 7). However, 19 conserved miRNAs and 2 novel miRNAs show abundant expression in leaf. 88 potential target genes were identified, including 29 coding unigenes and 59 non-coding unigenes. None of miRNAs showed negative correlation with their target unigenes, while some unigenes have positive correlation with miRNAs, such as GAMYB (targeted by miR159a, miR159c, and miR159d), EAF6 (targeted by miRNA156a) and receptor-like protein kinase At2g23200 (targeted by miR172b).

### Role of miRNAs in the development of stem

**Fig 8:**
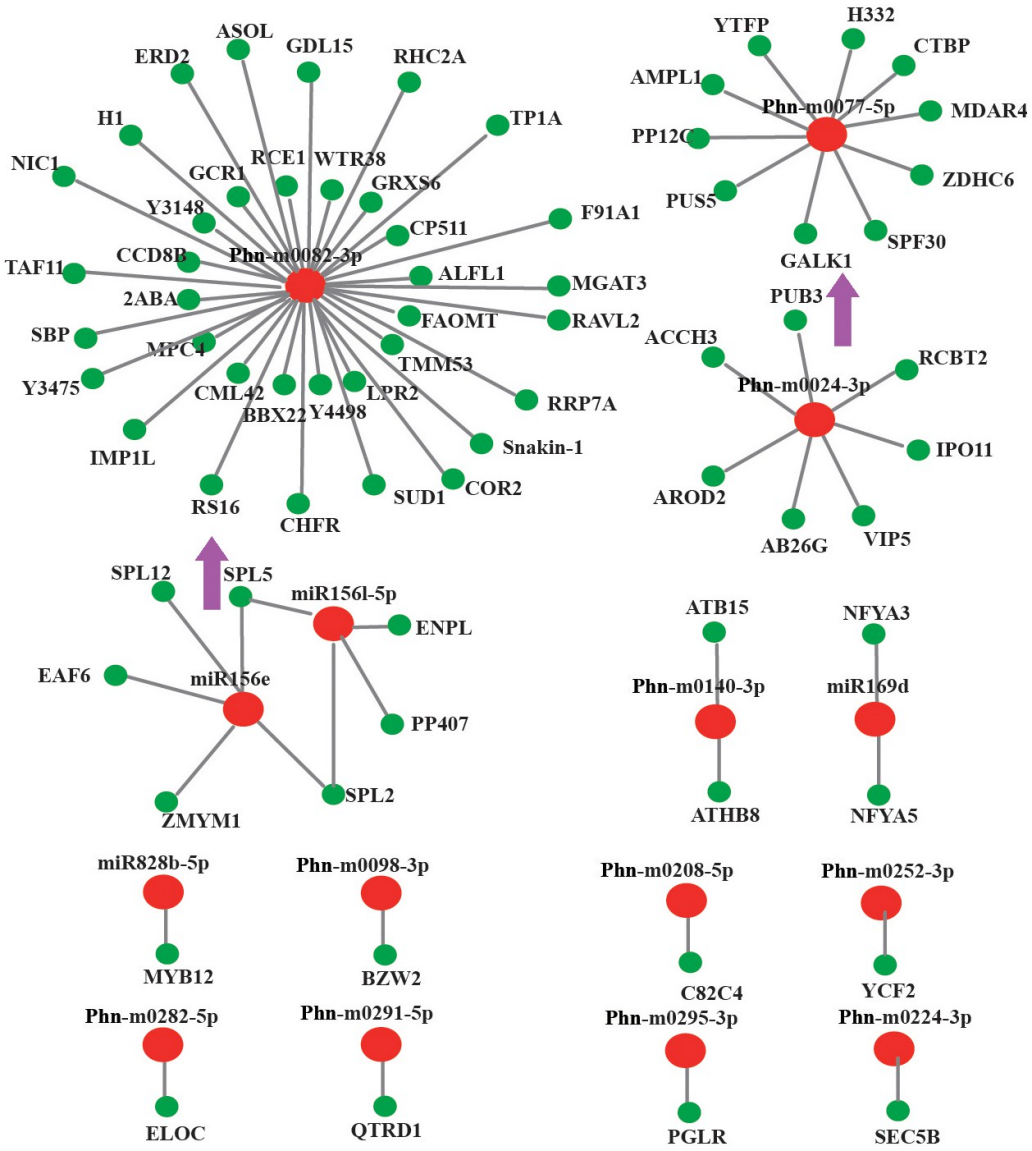
Stem-special miRNAs and their target genes. A combined view of stem-special miRNAs and their targets. Pink tip represents significant negative correlation.

3 conserved miRNAs and 12 novel miRNAs have abundance transcripts in stem (S11 Table). 239 potential unigenes targeted by 15 miRNAs were identified, including 75 coding mRNA and 164 non-coding mRNA. Interestingly, a few miRNAs have several potential target genes, such as 36 coding mRNA targeted by Phn-m0082-3p, 10 coding mRNA targeted by Phn-m0077-5p, and 7 coding mRNA targeted by Phn-m0024-3p. miR156l-5p and Phn-m0224-3p showed low expression in stem and high expression in underground tissues and leaf. The miR156l-5p showed significant negative correlation with SPL5 (Fig 8).

### Role of miRNAs in the development of tuberous root

**Fig 9:**
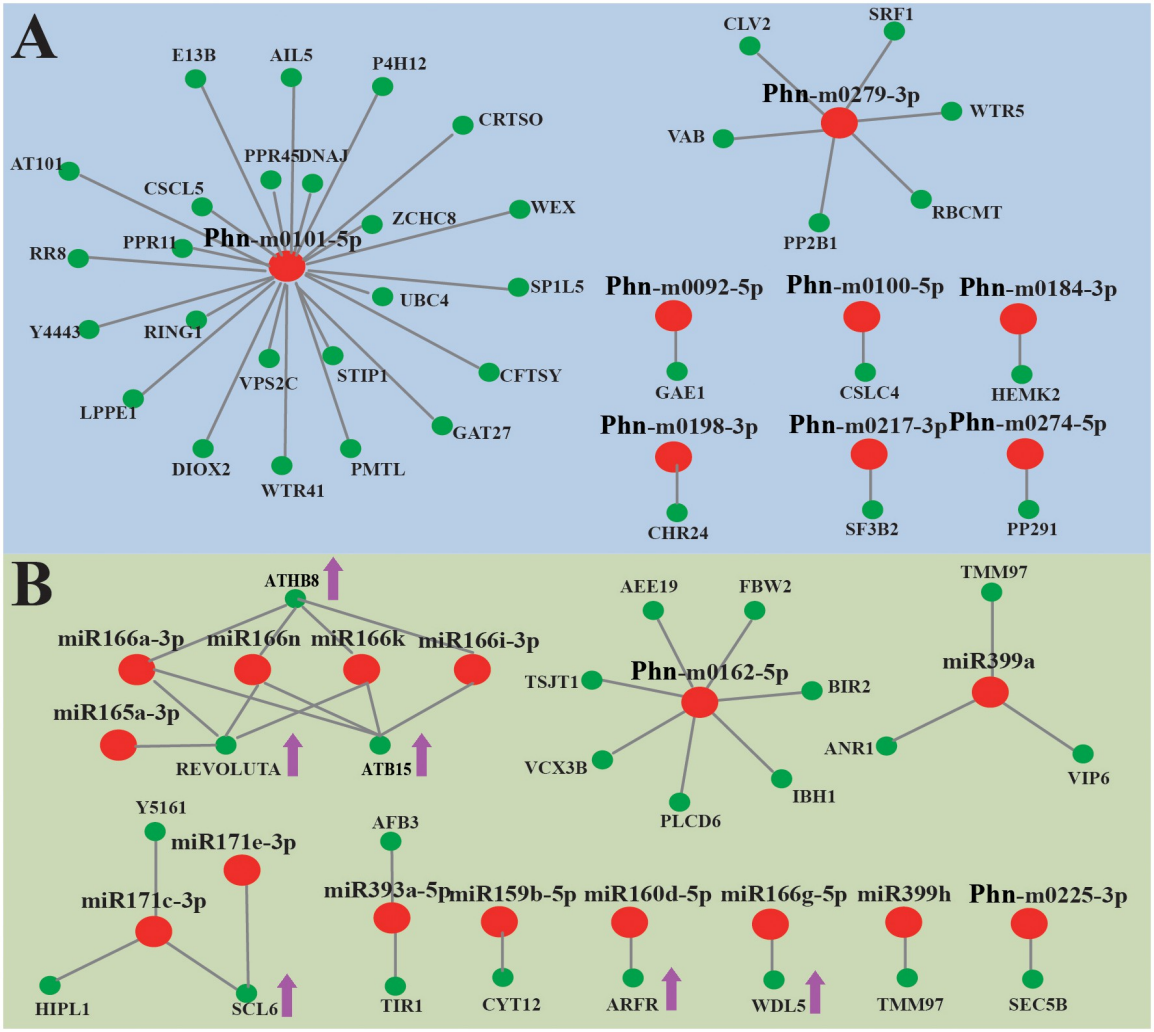
Xylem-special miRNAs in tuberous root and their target genes. (A) A combined view of up-regulated miRNAs in xylem of the tuberous root and their targets; (B) A combined view of down-regulated miRNAs in xylem of the tuberous root and their targets. Pink tip represents significant negative correlation between miRNAs and their target unigenes.

To explore the function of miRNAs in the development of tuberous root, tissue-special miRNAs in xylem and bark have been identified. 12 novel miRNAs were up-regulated in the root xylem (S12 Table). 96 potential target genes were identified, including 36 coding unigenes and 60 non-coding unigenes. Though 24 coding unigenes targeted by Phn-m0101-5p and 6 coding unigenes targeted by Phn-m0279-3p were observed, none of miRNAs showed negative correlation with their potential target unigenes (Fig 9). However, 16 conserved miRNAs and 5 novel miRNAs showed low expression in the root xylem. 77 potential target genes were identified, including 38 coding unigenes and 39 non-coding unigenes. Three HD-ZIP IIIs showed extreme negative correlation with the miR166 family. It seems that SCL6, BIR2, ARFR, and WDL5, were negatively regulated by the miR171 family, Phn-m0162-5p, miR160d-5p and miR166g-5p in xylem of the tuberous root, respectively. In addition, 7 conserved miRNAs were especially up-regulated in root bark (S13 Table), including 3 members of the miR160 family (miR160f-5p, miR160g and miR160h), 2 members of miR390 family (miR390-3p and miR390d-3p), miR169w and miR171a. However, none of miRNAs showed negative correlation with their potential target genes.

### Role of miRNAs in hormone signal transduction

The hormone plays a curial role in the morphogenesis of plant organs. In this study, we analyzed the regulation function of miRNAs on hormone signal transduction by searching their target genes. A large number of unigenes in hormone signal transduction pathways were targeted by identified miRNAs in *P*. *heterophylla*, mainly involving signal transduction of auxin, zeatin, abscisic acid, and jasmonic acid (S14 Table). Five gene families in the auxin signal transduction pathway may strongly be regulated by miRNAs from *P*. *heterophylla*, including TIR1, Aux/IAA, and ARF. SAUR was targeted by 10 miRNAs, including 2 known miRNAs and 8 novel miRNAs. In CTK signaling transduction, there were several binding sites of miRNAs in the sequences of AHP, B-ARR and A-ARR. Interestingly, PYL, PP2C and, SnRK2 in the ABA signal transduction pathway may strongly be regulated by miRNAs, mainly composed of novel miRNAs. We also found that a few novel miRNAs may regulate the expression of COI1 and JAZ in jasmonic acid signaling transduction. In addition, gibberellin receptor GID2 in gibberellin signal transduction was targeted by Phn-m0174-5p. BSK in the brassinosteroid signaling transduction pathway was targeted by 3 known miRNAs and 5 novel miRNAs. A few unigenes involving with the ethylene signal transduction were identified, such as MPK6 (targeted by Phn-m0087-5p, Phn-m0154-3p and Phn-m0281-5p), EBF1/2 (targeted by Phn-m0033-5p), and EIN3 (targeted by mir166e-5p and Phn-m0122-3p).

### Role of miRNAs in the interaction between plants and pathogens

**Fig 10:**
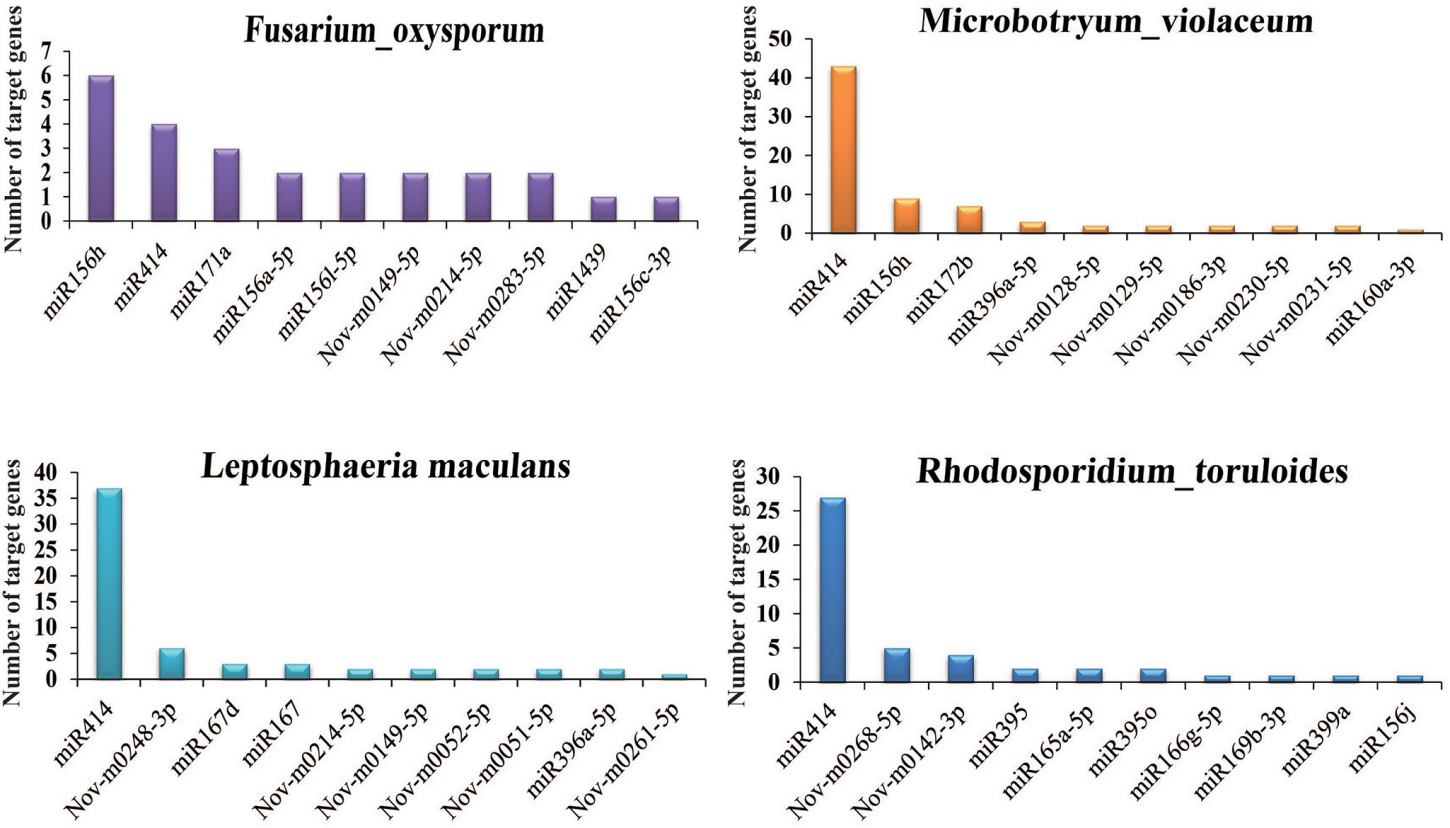
Number of genes from pathogens targeted by miRNAs from *P*. *heterophylla*.

For a better understanding of the role of miRNAs in the interaction between plants and pathogens, mRNA sequences of 4 microorganisms (*Fusarium oxysporum*, *Leptosphaeria maculans*, *Microbotryum violaceum*, *Rhodosporidium toruloides*) were downloaded from Genbank and used for searching the target genes of miRNAs from *P*. *heterophylla* (Fig 10 and S15 Table). 39 genes from *F*. *oxysporum* were targeted by 30 miRNAs of *P*. *heterophylla*, including 15 known miRNAs and 15 novel miRNAs. 6 genes were targeted by miR156h, followed by miR414 (4 genes) and miR171a (3 genes). Furthermore, some important genes of *F*. *oxysporum* may be regulated by miRNAs from *P*. *heterophylla*, such as pectin lyase F (targeted by Phn-m0104-3p), *30S* ribosomal protein S17-like protein (targeted by miR171e-3p) and myo-inositol transport protein ITR1 (targeted by miR171a). Interestingly, there were 37, 43, and 27 genes in *L*. *maculans*, *M*. *violaceum*, and *R*. *toruloides* targeted by miR414, respectively. These results suggested that miR414 may play an important role in the interaction between *P*. *heterophylla* and its pathogens.

### miRNA-induced cleavage of predicted targets

**Fig 11:**
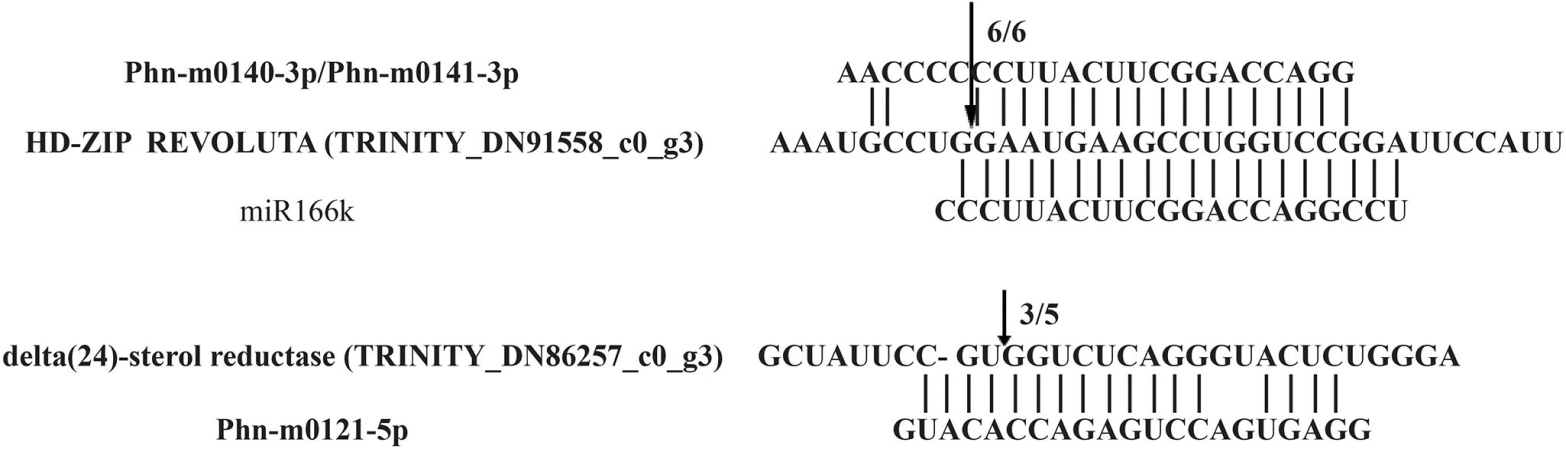
Cleavage sites identified by 5′ RLM-RACE. Cleavage site of targets unigenes were labeled by the arrows. Different fractions of cloned PCR products were labeled with the numbers.

The cleavage site of predicted targets of a conserved miRNA and a novel miRNA in *P*. *heterophylla* was further verified by 5′ RLM-RACE (Fig 11), respectively. HD-ZIP REVOLUTA and delta (24)-sterol reductase were targeted by miR166k and Phn-m0121-5p, respectively. All the fragments of 5′ RLM-RACE were analyzed on agarose gel, purified, cloned, and sequenced. Delta (24)-sterol reductase (TRINITY_DN86257_c0_g3) was found to be cleaved between 5 and 6. Interestingly, a new cleavage site of HD-ZIP REVOLUTA (TRINITY_DN91558_c0_g3) was found between G: G bases. This result suggested HD-ZIP REVOLUTA may be coordinately regulated by miR166k and Phn-m0140-3p/Phn-m0141-3p.

## Discussion

In earlier studies, a large numbers of miRNAs were identified from various plants through computational and direct cloning approaches from different tissues of certain periods [19–23], and plenty of them showed specifical expression similar to the coding genes. For example, 89 conserved miRNAs and 259 potato-specific miRNAs were discovered in root, stem, leaf, and tuber of potato [24] and a few of them demonstrated tuberization stage-specific expressions. In this study, 163 conserved miRNAs and 303 level miRNAs were identified and some of them also showed specifically up or down-regulated expression in leaf, stem, xylem and bark of the tuberous root. These results may help to reveal the role of miRNAs in the morphogenesis of different organs.

In fact, the morphogenesis of organs depends on expression pattern of genes in plants [6–8]. In this study, the expression patterns of miRNAs in different tissues were analyzed and these roles in organ morphogenesis have been revealed. Two members of miRNA172 were significantly up-regulated in the terrestrial tissues, and might be associated with curl symptoms of leaf and affect the flowering time [18]. A few members of miRNA164 family and miRNA169 family were significantly up-regulated in the underground tissues. miRNA164 may reduce the formation of lateral roots by down-regulating the expression level of *NAC1* [34]. miRNA169 may affect the number and size of certain cells in root meristem and the initiation of lateral roots [35], and participate in the response of roots to environmental stresses, such as drought and salt stress [36].

In reality, the formation of tuberous root mainly depends on the formation of secondary xylem from phloem and the expansion of parenchyma cells [37]. Therefore, the development regulation of xylem plays a crucial role in the formation of the tuberous root. In this study, several members of miR171, miR160, miR399, and miR166 families were significantly down-regulated in the xylem of tuberous roots of *P*. *heterophylla*. miR171 may affect the growth and development of tuberous roots in *P*. *heterophylla* by regulating three target genes of the scar-like family, namely SCL6-II, SCL6-III, and SCL6-IV [38, 39], controlling the radial growth of roots [40] and hormone signal [41]. miR166 may affect the number of lateral roots and symbiotic nodules, and induce the ectopic development of vascular bundles in these transgenic roots [42]. In addition, 12 novel miRNAs were significantly up-regulated in the xylem of tuberous root, which suggested its important role in the development of root xylem.

miRNA not only directly regulates the development of plants through its target genes, but also indirectly affects the morphogenesis of organs by regulating the expression of genes involved in hormone signal transduction. Previous studies have shown that ABA, CTK, and JA increased gradually with the development of tuberous roots in *P*. *heterophylla* [43], and which involved in the regulation of cell proliferation, cell elongation, cell expansion [44], formation of microtubule microfilaments [45] and accumulation of assimilates in tuberous roots [46]. In this study, several key genes in hormone signal transduction were targeted by miRNAs from *P*. *heterophylla*, such as miR393 and miR172. Interestingly, most of unigenes involved in signal transduction of ABA, CTK, and JA were targeted by novel miRNAs, which suggested a different regulation mechanism of miRNAs from *P*. *heterophylla*.

Many plants have evolved perfect defense mechanisms, including the miRNA system. miR393 was firstly discovered and regulated by necrotroph pathogens [47]. A variety of new miRNAs could be detected in plants infected by the rice stripe virus [48, 49]. In our previous studies, five kinds of pathogens were identified in *P*. *heterophylla*, in which *M*. *violaceum*, *L*. *maculans*, and *P*. *fluorescens* are typical harmful bacteria [29]. In this study, a large number of mRNAs from four kinds of pathogens were targeted by miRNAs from *P*. *heterophylla*. Several genes were targeted by miR414, while none of unigenes in *P*. *heterophylla* were targeted. These results suggested that miR414 may provide a new way to overcome the disease resistance and continuous cropping obstacles of plants. However, miR414 showed extremely low expression in the tissue of *P*. *heterophylla* and it is necessary to clarify its function in the defense of pathogens.

## Conclusions

In this study, we reported a population of *P*. *heterophylla* miRNAs from four different vegetative tissues. A total of 163 conserved miRNAs and 303 level miRNAs were identified and some of them showed specifically up or down-regulated expression in different tissues. Some unigenes involved in Plant-pathogen interaction, MAPK signaling pathway-plant and signal transduction of auxin, zeatin, abscisic acid and jasmonic acid were targeted by miRNAs from *P*. *heterophylla*. A large number of mRNAs of four pathogens were targeted by miRNAs from *P*. *heterophylla* and several genes were targeted by miR414. Predicted target genes of a conserved and novel miRNA were validated by 5′ RLM-RACE. These results may help to explain the function of miRNAs in the morphogenesis of organs and defense of pathogens, and provide theoretical basis for breeding and genetic improvement of *P*. *heterophyll*a.

## Materials and methods

### Plant material and total RNA isolation

*P*. *heterophylla* plants (Shitai NO.1) were selected by our group and certified as a national variety by Guizhou Crop Variety Approval Committee in 2016 (Accession number: 2016002). It has become a very popular variety in Guizhou Province because of its excellent quality and high yield. Plants were grown in the experimental field of Shibing County, Guizhou Province, China. The leaves, stem, and tuberous root (cortex and xylem) of *P*. *heterophylla* were collected separately from three randomly selected individuals. After cleaning, all samples were immediately frozen in liquid nitrogen. Transzol Plant RNA Extraction Kit (TaKaRa, Tokyo, Japan) was used for the extraction of total RNA according to the manufacturer’s instructions. Traces of remaining DNA were removed by using DNase I (Takara, Tokyo, Japan).

### cDNA library preparation and small RNA library construction

For a better understanding of miRNAs and mRNAs networks in *P*. *heterophylla*, the cDNA library of these tissues was also constructed. The total RNA of four tissues was purified by using Oligo(dT) magnetic beads, respectively. The mRNA was disrupted into small fragments and used for second-strand cDNA synthesis. These cDNA fragments were ligated with the Illumina paired-end sequencing adaptors and sequenced by using the Illumina Hiseq4000 platform. The expression level of each gene in different tissues was normalized by using the reads per kb per million reads (RPKM) and the RPKM of each unigene refers to the sum of RPKM from all isoforms of the same gene.

Small RNA libraries for each sample were prepared independently using Truseq TM Small RNA sample prep Kit (Illumina, U.S.A). Total RNAs of *P*. *heterophylla* were firstly size-fractioned on 15% denaturing polyacrylamide gel and small RNAs (18–35nt) fraction was collected. Next, T4 RNA ligase was used to ligate the small RNAs and adapters (5′and3′). Small RNAs with adapters were reverse-transcribed using SuperScript II Reverse Transcriptase (Invitrogen, U.S.A) and PCR amplified with PCR. The amplification products were excised from the Novex 6% TBE-Urea Gel. The cDNA samples were sequenced using Illumina Hi-seq2000 at Shanghai Majorbio Bio-pharm Technology Co., Ltd. (Shanghai, China). To obtain the clean reads, reads with low-quality reads, adaptor sequences, and the reads shorter than 18nt and longer than 30nt were removed.

### Identification of miRNAs and differential expression analysis of miRNAs

These high-quality sRNA sequences were mapped to the mRNA transcriptome database. These perfectly mapped sRNAs were used for further analysis, while rRNA, tRNA, small nuclear (snRNA), and small nucleolar RNA (snoRNA) were removed. The remaining sRNAs were aligned with the mature miRNA or miRNA precursor from all plant species in the miRBase Sequence Database (http://www.mirbase.org/, release 22.1, October 2018) with the allowance of two mismatches. These perfectly matched sRNAs were collected and considered to be conserved miRNAs. Potentially novel miRNAs were predicted using MIREAP (http://sourceforge.net/projects/mireap/), and the secondary structure of the sRNA precursor was calculated by using the RNAfold web server [32] (http://rna.tbi.univie.ac.at/cgi-bin/RNAfold.cgi) with default parameters. Those sRNAs fitting all the published criteria and with no homologous sequence in the public databases were considered to be a putative novel miRNA precursor. The expression level of miRNAs was normalized to one million by total clean reads of miRNAs in each sample sequence. For the novel miRNAs, the names from Phn-miR1 to Phn -miR303 were given.

TPM (Transcripts per million) was used for identifying differentially expressed miRNAs in different tissues. When log FC (log fold expression change) was greater than 1 or less than -1 (FDR<0.001, *P*-value<0.005), miRNAs were considered to be differentially expressed.

### Prediction of miRNA targets

The prediction of miRNA targets was performed by using the Target Finder (https://github.com/carringtonlab/TargetFinder) against the transcriptome sequence data of *P*. *heterophylla* in previous study. Maximum expectation and complementarity scoring were used for evaluating the miRNA-target mRNA interactions according to the method described by Allen et al [19].

### GO and KEGG analysis

For a better understanding of the function of the target genes and their corresponding metabolic network regulated by these miRNAs, Gene Ontology (GO) and the Kyoto Encyclopedia of Genes and Genomes (KEGG) were used. All dates were corrected by Bonferroni. When the corrected p-value (FDR) is less than 0.05, it is considered that the GO or KEGG function is significantly enriched.

### Co-expression analysis of miRNAs and their target genes

To analyze the regulation of miRNAs and their target genes, the expression level of Spearman correlation coefficients between the miRNAs and their target genes were performed.

### Target validation of miRNAs by 5′ RLM-RACE

The cleavage site of predicted targets was determined by using a 5′RLM-RACE (Takara, Tokyo, Japan) according to the instructions of the manufacturer. Briefly, the RNA adapter was ligated to the mRNA using the T4 RNA ligase and reverse transcribed to cDNA. Gene-specific reverse primers and 5eadapter primers were used to amplify the cleaved transcripts. The primers used to amplify cleavage products of miRNA target genes in *P*. *heterophylla* are listed in the S16 Table. The amplifying fragments were cloned to pMD19-T vectors (Takara, Tokyo, Japan) and sequenced by M13 primers.

## Supporting information

S1 Table Conserved miRNAs. (XLS)

S2 Table Detailed information of identified novel miRNAs. (XLSX)

S3 Table Target genes of conserved miRNAs. (XLSX)

S4 Table Target genes of novel miRNAs. (XLSX)

S5 Table Unigenes targeted by some miRNAs. (XLSX)

S6 Table GO analysis of predicted target genes of miRNAs. (XLS)

S7 Table KEGG analysis of predicted target genes of miRNAs. (XLS)

S8 Table Expression correlation analysis between miRNAs and their target genes. (XLS)

S9 Table Up-regulated miRNAs and their target genes in terrestrial tissues and underground tissues. (XLSX)

S10 Table Up-regulated and down-regulated miRNAs and their target genes in leaf. (XLSX)

S11 Table Up-regulated miRNAs and their target genes in stem. (XLSX)

S12 Table Up-regulated and down-regulated miRNAs and their target genes in xylem of the tuberous root. (XLSX)

S13 Table Up-regulated miRNAs and their target genes in bark of the tuberous root. (XLSX)

S14 Table miRNAs and target genes in the biosynthesis of plant hormones. (XLSX)

S15 Table Genes from four pathogens targeted by miRNAs. (XLSX)

S16 Table Primers used to amplify cleavage products of miRNA target genes through 5’ RLM-RACE. (XLSX)
